# Evidence for the Application of Emerging Technologies to Accelerate Crop Improvement – A Collaborative Pipeline to Introgress Herbicide Tolerance Into Chickpea

**DOI:** 10.3389/fpls.2021.779122

**Published:** 2021-12-03

**Authors:** Janine Croser, Dili Mao, Nicole Dron, Simon Michelmore, Larn McMurray, Christopher Preston, Dylan Bruce, Francis Chuks Ogbonnaya, Federico Martin Ribalta, Julie Hayes, Judith Lichtenzveig, William Erskine, Brian Cullis, Tim Sutton, Kristy Hobson

**Affiliations:** ^1^School of Agriculture and Environment, The University of Western Australia, Perth, WA, Australia; ^2^Institute of Agriculture, The University of Western Australia, Perth, WA, Australia; ^3^South Australian Research and Development Institute, Adelaide, SA, Australia; ^4^School of Agriculture, Food and Wine, The University of Adelaide, Adelaide, SA, Australia; ^5^Tamworth Agricultural Institute, New South Wales Department of Primary Industries, Tamworth, NSW, Australia; ^6^Global Grain Genetics, Clare, SA, Australia; ^7^Grains Research and Development Corporation, Canberra, ACT, Australia; ^8^Centre for Biometrics and Data Science for Sustainable Primary Industries, University of Wollongong, Wollongong, NSW, Australia

**Keywords:** mutation, imidazolinone, sulfonylurea, marker-assisted selection (MAS), accelerated single seed descent, sparse phenotyping

## Abstract

Accelerating genetic gain in crop improvement is required to ensure improved yield and yield stability under increasingly challenging climatic conditions. This case study demonstrates the effective confluence of innovative breeding technologies within a collaborative breeding framework to develop and rapidly introgress imidazolinone Group 2 herbicide tolerance into an adapted Australian chickpea genetic background. A well-adapted, high-yielding desi cultivar PBA HatTrick was treated with ethyl methanesulfonate to generate mutations in the *ACETOHYDROXYACID SYNTHASE 1* (*CaAHAS1*) gene. After 2 years of field screening with imidazolinone herbicide across >20 ha and controlled environment progeny screening, two selections were identified which exhibited putative herbicide tolerance. Both selections contained the same single amino acid substitution, from alanine to valine at position 205 (A_205_V) in the AHAS1 protein, and KASP™ markers were developed to discriminate between tolerant and intolerant genotypes. A pipeline combining conventional crossing and F_2_ production with accelerated single seed descent from F_2:4_ and marker-assisted selection at F_2_ rapidly introgressed the herbicide tolerance trait from one of the mutant selections, D15PAHI002, into PBA Seamer, a desi cultivar adapted to Australian cropping areas. Field evaluation of the derivatives of the D15PAHI002 × PBA Seamer cross was analyzed using a factor analytic mixed model statistical approach designed to accommodate low seed numbers resulting from accelerated single seed descent. To further accelerate trait introgression, field evaluation trials were undertaken concurrent with crop safety testing trials. In 2020, 4 years after the initial cross, an advanced line selection CBA2061, bearing acetohydroxyacid synthase (AHAS) inhibitor tolerance and agronomic and disease resistance traits comparable to parent PBA Seamer, was entered into Australian National Variety Trials as a precursor to cultivar registration. The combination of cross-institutional collaboration and the application of novel pre-breeding platforms and statistical technologies facilitated a 3-year saving compared to a traditional breeding approach. This breeding pipeline can be used as a model to accelerate genetic gain in other self-pollinating species, particularly food legumes.

## Introduction

Achieving food security under increasingly hostile environmental conditions^1^ requires rapid innovation across all sectors involved in food production (Varshney et al., 2021). Effectively harnessing pre-breeding tools will be key to improving the rate of genetic gain in crop and horticultural species. For more than two decades, tools such as diagnostic markers for trait selection have improved the efficiency of plant breeding programs (Eagles et al., 2001; Xu et al., 2017). More recently, techniques to accelerate lifecycle turnover using modified single seed descent have been proposed to further truncate breeding pipelines in a range of species, including food legumes (Ochatt et al., 2002; Mobini et al., 2015; Croser et al., 2016; Mobini and Warkentin, 2016; Ribalta et al., 2017; Watson et al., 2018; Hickey et al., 2019; Cazzola et al., 2021). Missing from the literature is evidence of how these complementary established and emerging pre-breeding techniques can be combined and harnessed in a genetic improvement program to reduce the time to variety release. For food legumes, genetic improvement programs are generally in the public domain and under-resourced compared to cereals and oilseeds. As a result, breeding programs need to be agile to maximize efficiency involving collaboration with public pre-breeding at other institutions. Here, we present a case study of cross-institutional implementation of a compressed breeding pipeline to deliver an improved chickpea (*Cicer arietinum* L.) cultivar bearing a mutagenesis-derived gene for herbicide tolerance, a critical need of Australian grain growers for sustainably managing their farming system and improving production.

Among emerging pre-breeding techniques, the reduction of generation cycle time is the most cost-effective way to increase the rate of genetic gain (Cobb et al., 2019). Rate of genetic gain is measured by the breeders equation (Lush, 1943) where generation cycle time is the denominator and thus any time-saving innovation is beneficial to the overall rate of gain (Li et al., 2018). For chickpea, an accelerated single seed descent (aSSD) platform (modified from Croser et al., 2016) has been applied within the Australian breeding program on a commercial scale since 2015. Based on the principles of single seed descent (Goulden, 1939; Brim, 1966), plants are grown under controlled conditions designed to reduce vegetative biomass partitioning and prioritize reproductive speed. A combination of photoperiod extension, late-spring temperatures, light wavelength optimization, and precocious germination technology enable 50 to 60-day lifecycle completion year-round for chickpea, irrespective of the field flowering phenology (Croser et al., 2016; Atieno et al., 2021). Harnessing out-of-season accelerated generation turnover in combination with complementary tools such as marker-assisted selection (MAS) enables rapid identification and culling of lines not homozygous for a desired trait. The combined aSSD-MAS approach is thus a highly efficient method to rapidly introgress novel traits and target downstream breeding program resource allocation and forms a key role in the compressed breeding pipeline described here for chickpea.

Chickpea is grown across more than 14 million ha worldwide (Bulti and Haji, 2019). India is the largest producer and Australia, with up to one million ha under cultivation, comes in second (FAOSTAT, 2021). Expansion of the Australian chickpea production area has been driven by improved varieties. Such varieties deliver up to 85 kg of nitrogen per hectare (Herridge et al., 1995) and provide a financially viable crop to disrupt cereal fungal diseases such as “take all” (caused by *Gaeumannomyces graminis var. tritici*) and “crown rot” (caused by *Fusarium pseudograminearum*) (Kirkegaard et al., 2008). A major barrier to further uptake of chickpea is its poor competitiveness against common weed species and lack of in-crop weed control options. Australian producers wanting to incorporate chickpea into rotations are also deterred by its sensitivity to commonly used acetohydroxyacid synthase (AHAS)-inhibitor herbicides (Group 2), popular in Australian farming systems due to their broad-spectrum efficacy. The use of AHAS inhibitors to control weeds in other crops can prevent the inclusion of chickpea in a rotation for 2 years or longer in dry environments with a soil pH > 6.5 (Hollaway et al., 2006).

The problem of susceptibility to residual herbicides is unlikely to be overcome by new herbicide chemistries due to regulatory and cost barriers. As a result, the development of herbicide-tolerant (HT) pulse crops is seen as a sustainable management option to enable existing herbicides to be used in new ways (Rüegg et al., 2007). Group 2 herbicide tolerance has been identified in Canadian chickpea germplasm (Thompson and Tar’an, 2014), however, these varieties are not adapted to Australian growing conditions. Mutagenesis has been particularly successful in developing tolerance to AHAS-inhibitor herbicides (Tan et al., 2005; Green, 2014), and the trait is now available in crops including maize (*Zea mays* L.), wheat (*Triticum aestivum* L.), canola (*Brassica napus* L.), barley (*Hordeum vulgare* L.), lentil (*Lens culinaris* Medik.), Sorghum (*Sorghum bicolor* (L.) Moench.), and sunflower (*Helianthus annuus* L.) (Tan et al., 2005; Tuinstra and Al-Khatib, 2008; Lee et al., 2011). AHAS inhibitors target the AHAS enzyme which catalyzes the first step of branched chain amino acid synthesis. Target-site tolerance is well described in weed and crop species and can be conferred by single amino acid residue substitutions at any of eight conserved sites in the AHAS protein (Duggleby et al., 2008). For chickpea, development of target-site tolerance to AHAS-inhibitor herbicides and incorporation of the trait into a well-adapted genetic background suited to Australian production would increase in-crop weed control options and reduce plant-back restrictions from soil residues of herbicides applied in previous crops.

If herbicide tolerance can be identified there remains the challenge to introgress the trait as quickly as possible into a well-adapted genetic background, retaining other key production traits such as resistance to Ascochyta blight (caused by *Ascochyta rabiei*). Rapid introgression of any trait is reliant on access to techniques to speed up conventional breeding timelines, as well as a method for statistically assessing superior genotypes among low seed numbers produced by rapid generation cycling techniques. Much of the challenge associated with selection of superior genotypes for yield is due to the magnitude of the genotype by environment interaction (GEI) (Cullis et al., 1996, 2010). The genetic value of genotypes for grain yield is predicted using data obtained from variety trials grown across multiple environments being representative of the target production environments. A multi-environment trial (MET) is a collection of variety trials conducted over a range of geographic locations and years and a growing body of literature illustrates the advantages in analyzing yield data from MET datasets using a factor analytic linear mixed model (FALMM) (e.g., Oakey et al., 2007, 2016; Beeck et al., 2010; Gogel et al., 2018). While FALMM has traditionally been used for large MET field datasets, a major advantage of this approach is the ability to accommodate incomplete MET data, i.e., not all varieties grown at all environments in a model-based approach. Here, the FALMM statistical approach is adapted to identify elite lines despite low seed numbers coming from aSSD and other controlled environment screening techniques, without compromising the speed of advanced line progression through the breeding pipeline.

Within the context of access to well-established protocols to improve breeding efficiency in chickpea, we set out to determine the feasibility of integrating, across public institutions, established pre-breeding and emerging breeding tools into a single, compressed crop-improvement pipeline. We describe the use of mutagenesis to develop a non-GM herbicide tolerance trait beneficial within the farming system. We provide a case study outlining the progression, in 4 years, from initial cross to Australian National Variety Trials (NVT) of herbicide-tolerant chickpea breeding line CBA2061 using a combination of innovative platforms: accelerated homozygosity, marker-assisted selection and advances in applied statistics optimal for achieving selection targets. We outline the effectiveness of efficient yield evaluation designs to fast-track entry into advanced breeding trials. In doing so, we provide evidence of a successful, comprehensive, and collaborative approach to accelerate genetic gain in chickpea which can be modified for trait introgression in other self-pollinating species.

## Materials and Methods

### Breeding Approach, Germplasm, and Locations

**FIGURE 1:**
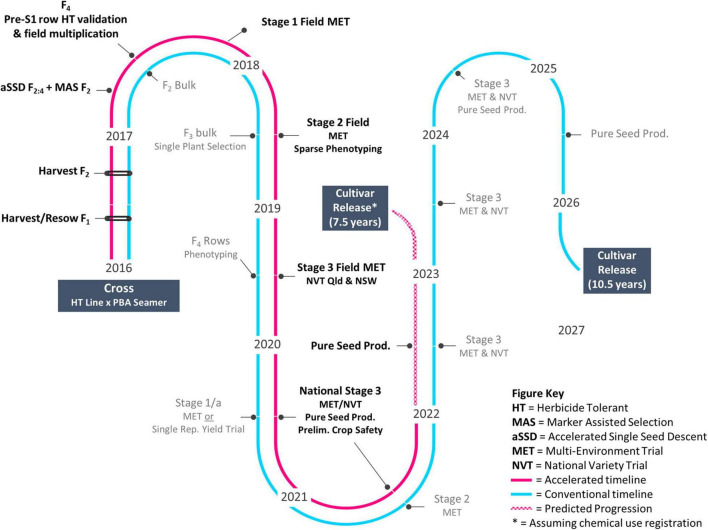
Accelerated vs. conventional trait introgression timeline.

Research activities and field trials as shown in Figure 1 were undertaken across five publicly funded organizations (Table 1). Germplasm was provided by SARDI and the NSW DPI breeding program (Table 2).

**TABLE 1:** Collaborating institutions, locations, and roles.

Institution	Role in accelerated breeding pipeline
South Australian Research and Development Institute* (SARDI)	Group 2 HT^†^ development (SARDI_UoA) KASP marker development and application CE^††^ Ascochyta blight screening Crop safety field trials
University of Adelaide (UoA)	HT trait development (SARDI_UoA) Crop safety field trial design/interpretation
New South Wales Department of Primary Industries* (NSW DPI)	Pulse Breeding Australia** lead – hybridization, yield and herbicide field trials, herbicide selection screening. Field Ascochyta blight and Phytophthora root rot (Tamworth, NSW and Warwick, QLD) screening. Herbicide validation trials.
University of Western Australia* (UWA)	Accelerated single seed descent
University of Wollongong (UoW)	Biometric designs for yield trials and MET analysis of sparse phenotyping

*Pulse Breeding Australia (PBA) partners. ^†^Herbicide tolerant, ^††^controlled environment. **Pulse Breeding Australia (PBA) chickpea program was a collaboration between Australian state-based departments of agriculture and The Grains Research and Development Corporation (GRDC).

**TABLE 2:** Genotypes, pedigree, and use of germplasm.

Genotype* Seed source	Pedigree	Use
PBA HatTrick NSW DPI	Descendant of a Jimbour/ICC14903 cross	M_0_ seed for mutation. Field and CE^†^ studies.
D15PAHI002 SARDI	PBA HatTrick mutant with tolerance to HRAC Group 2 herbicide	Mutation line, identified at M_3_ Female parent of CBA2061
D16PAHI001 SARDI	PBA HatTrick mutant with tolerance to HRAC Group 2 herbicide	Mutation line, identified at M_3_
PBA Seamer NSW DPI	Descendant of a 98081-3024/PBA HatTrick cross	Pollen parent of CBA2061 Field and CE^†^ studies.
CBA Captain NSW DPI	Australian cultivar, descendant of a CICA0910/D06314 > F3BREE2AB014 cross	Field and CE^†^ studies.
Kyabra NSW DPI	Australian cultivar, descendant of an Amethyst//94631/Barwon//Lasseter/940-26//946-31/Norwin//8507-28H//Amethyst//T1069/8507-28H//946-31 cross	Field and CE^†^ studies.
Genesis090* NSW DPI	Australian cultivar, descendent of FLIP82-150C/FLIP83-48C	Field and CE^†^ studies.
PBA Boundary NSW DPI	Australian cultivar, descendant of JIMBOUR/ICC3996	Field studies.
PBA Seamer NSW DPI	Australian cultivar, descendent of 98081-3024/PBAHATTRICK	Field and CE^†^ studies.
PBA Slasher NSW DPI	Australian cultivar, descendent of HOWZAT/ICC3996	Field and CE^†^ studies.
PBA Magnus NSW DPI	Australian cultivar, descendent of FLIP97-159C/MACARENA//GENESIS114	Field and CE^†^ studies.
CBA2061 NSW DPI	Advanced breeding line derived from cross between M_4_ D15PAHI002 × PBA Seamer National Variety Trial entry	Prospective HT cultivar.

*Kabuli-type cultivar, all other germplasm: desi-type. ^†^ Controlled environment.

### Seed Mutation and Selection of Genotypes With Tolerance to Acetohydroxyacid Synthase-Inhibitor Herbicides

In 2013, a sample of c. 100,000 seeds (M_0_) of PBA HatTrick were treated at SARDI with ethyl methanesulfonate (EMS) following the method of Mao et al. (2019). All seed multiplication and herbicide selection trials were undertaken at a site near Paskeville, South Australia. In 2013/14, the seed was field-bulked. In 2015, 1500 kg of M_3_ seed was sown across 20 ha and screened with imazapyr herbicide at 300 g active ingredient (a.i.) ha^–1^ (Unimaz ^®^ 250 g L^–1^ imazapyr, UPL Australia Ltd). Six weeks after herbicide application, M_3_ phenotypic selections were made based on the absence of visual herbicide damage symptoms such as leaf chlorosis, necrosis, or plant stunting. Selections were transplanted prior to flowering into 13.5 L plastic pots and grown in a shade house with hand-watering until harvest of M_4_. For progeny testing, 10 M_4_ seeds per selection were sown into 0.47 L plastic pots and at the 4–5 node stage, hand-sprayed with 37.5 g a.i. ha^–1^ of imazapyr to confirm herbicide tolerance (lethal rate for PBA HatTrick determined by preliminary pot test – data not shown). Herbicide damage was visually assessed 21 days after treatment (DAT).

In 2016, 80 kg of the same M_2_ seed was sown across 1 ha and screened with 105 g of imazapic + 35 g of imazapyr a.i. ha^–1^ (Onduty ^®^ 525 g kg^–1^ of imazapic + 175 g kg^–1^ of imazapyr, BASF Australia Ltd). The M_2_ selections were transplanted to 13.5 L pots as described above and M_3_ seeds were harvested. Ten seeds from each selection were progeny-tested using 13 g of imazapic + 4.4 g of imazapyr ha ^–1^ (lethal rate for PBA HatTrick determined by preliminary pot test – data not shown). Herbicide damage was visually assessed 21 DAT.

### Herbicide Dose Response Experiment

The two mutant lines D15PAHI002 (identified in 2015) and D16PAHI001 (identified in 2016) were confirmed with KASP™ markers to contain the A_205_V mutation. In 2018, D15PAHI002, D16PAHI001, and cv. PBA HatTrick were grown in 0.47 L pots in a randomised complete block design (RCBD) with four replicates. Plants were compared for their response to increasing rates of two AHAS-inhibitor herbicides imazapyr and chlorsulfuron, representing Group 2 families imidazolinone and sulfonylurea. Herbicide treatments were applied at the 4–5 node growth stage according to Mao et al. (2019). For each sample, above ground biomass was harvested 21 days after treatment (DAT), oven-dried at 60°C for 48 h, and dry weight was recorded. Data were analyzed using non-linear log-logistic regression models with the DRC package 3.0–1 in R v3.2.2.27 (R Core Team, 2020) as per Mao et al. (2019).

### Candidate Gene Information and Marker Development

Genomic DNA (gDNA) was extracted from the progeny of the two mutant lines and PBA HatTrick using a modified CTAB DNA extraction protocol (Doyle and Doyle, 1987). Primers described by Thompson and Tar’an (2014) were used to amplify the *CaAHAS1* gene. PCR reactions were purified using Nucleofast filter plates (Macherey-Nagel, Duren, Germany) and Sanger-sequenced. Sequence reads were compared against the CDC Frontier *CaAHAS1* sequence (Varshney et al., 2013) and single nucleotide polymorphisms (SNPs) were identified using Geneious 10.2^2^. Translated coding sequences were aligned to the *Arabidopsis thaliana* AHAS1 amino acid sequence (GenBank accession NM_114714.3). The chickpea AHAS1 protein mutations are referred to by their homologous positions in the *A. thaliana* sequence.

### Introgression of Target-Site Tolerance to Acetohydroxyacid Synthase-Inhibitor Herbicides Into Adapted Germplasm

Crossing between mutant M_5_ line D15PAHI002 and cv. PBA Seamer was undertaken at NSW DPI in a temperature-controlled glasshouse in March 2016. A key component to accelerated trait delivery was the provision of D15PAHI002 to the breeding program in early 2016, prior to full trait confirmation and we report only results related to derivatives of D15PAHI002. Parental lines were grown, and homozygosity of the *CaAHAS1* HT allele in parent plants was confirmed using MAS. Crossing was undertaken similar to that described by Kalve and Tadege (2017). Hybrid F_1_ seeds were grown and *n* = 46 F_2_ lines were produced. Two further populations were developed within the pipeline; D15PAHI002 crossed with a high-performing desi with Ascochyta blight resistance (D1007 > 11F2TMWR2SS007) and a high-performing kabuli (K15195 > F103). Additional F_2_ seeds developed from these cross combinations were split with one set (total *n* = 186, including the 46 lines from D15PAHI002 × PBA Seamer) couriered to UWA for accelerated single seed descent (aSSD) F_2:4_ while the other (*n* = 790) was kept at NSW DPI for Group 2 herbicide screening.

### Accelerated Single Seed Descent (aSSD) F_2:4_

F_2_ seeds (*n* = 186 individuals) were received at UWA in October 2016 for accelerated single seed descent (aSSD) as applied in Atieno et al. (2021). Two weeks after emergence, leaves were sampled and couriered to SARDI for MAS. Plants homozygous for the HT trait were retained and staked, and immature seeds were removed at physiological maturity, *c*. 18 days after flowering. In-pod immature seeds were dried in seed envelopes in a *c*. 3 cm bed of orange indicator silica gel for 5–7 days at 25°C. At 8% seed moisture, measured with an active water meter (Rotronics) and converted to a seed moisture reading based on a moisture sorption isotherm for chickpea (Menkov, 2000), seeds were resown to soil and grown under the same conditions as the F_2_ generation. The F_4_ seed were left for an additional 14 days post-physiological maturity on the parent plant and lines were couriered to NSW DPI in March 2017 for further multiplication and evaluation.

### Perlite-Based Controlled Environment Phenotyping for Tolerance to Acetohydroxyacid Synthase-Inhibitor Herbicides

A second set of F_2_ (*n* = 790 individuals) seeds were phenotyped for HT in a plastic house at NSW DPI. The F_2_ seedlings and parental checks were grown in slotted plastic trays containing perlite and, at the two-leaf growth stage, submerged for 10 s in a 2 ppm Imazapyr solution (Rotary Max ^®^ 240). Trays were drained and not watered for 24 h. At 48 h after treatment, plants displaying yellowing or wilting were discarded and tolerant plants were transplanted into 3.8 L pots. Leaves were sampled and couriered to SARDI for MAS. Homozygous HT plants were grown to maturity and F_3_ seeds were harvested.

### Marker-Assisted Selection Protocol

A KASP™ marker for the SNP identified in *CaAHAS1* was designed using the Kraken™ software package (LGC Biosearch Technologies, Middlesex, United Kingdom), and its reliability was confirmed on parental DNA samples. Genomic DNA was extracted and diluted to ∼5 ng μL^–1^, and ∼25 ng was used in KASP™ reactions using the LGC Genomics SNP Line system (LGC Biosearch Technologies, Middlesex, United Kingdom). Genotype calls were reported back to researchers at UWA and NSW DPI to select F_2_ lines homozygous for the HT allele for further progression through the aSSD pipeline.

### Herbicide Trait Confirmation, Yield, and Agronomic Evaluation

Line D15PAHI002, and 14 derivative HT sister F_4_ aSSD lines were sown into a trait confirmation row trial at NSW DPI in September 2017 and compared for herbicide response to conventional chickpea varieties PBA Seamer, PBA Slasher, PBA Magnus, and Genesis090. Imazapyr 240 g L^–1^ (Rotary Max ^®^ 240) was applied at the 6–8 node stage at a rate of 200 g ha^–1^. Tolerance was visually assessed 4 weeks after application, with symptoms progressing rapidly in warm spring conditions. In December 2017, lines were hand-harvested and machine-threshed for subsequent yield evaluation.

Seeds of HT line CBA2061 from the 2017 trait confirmation row trial were sown in a Stage 1 MET dataset and subsequent yield evaluation trials (Table 3; Figure 1). CBA2061 was assessed for agronomic suitability and yield against regionally specific cultivars CBA Captain, PBA HatTrick, PBA Boundary, PBA Seamer, and Kyabra. At each evaluation stage, plots were assessed for early vigor, flowering, maturity, plant height, and lodging. Lines were also assessed for resistance to Phytophthora root rot (caused by *Phytophthora medicaginis*) as per Bithell et al. (2021), and to locally aggressive isolates of Ascochyta blight (caused by *Ascochyta rabiei*) in controlled environment and field nurseries. Grain yield measurements were recorded at physiological maturity for each plot.

**TABLE 3:** NSW DPI breeding program yield trials, National Variety Trials (NVT), and pure seed initiation for prospective cultivar CBA2061.

Year	2018	2019	2020	2021
**Stage**	1 (F3:5)	2 (F3:6)	3	3
**Objective**	Seed bulk, MET*	MET	MET, NVT**	MET, NVT Pure seed initiation
**Number of sites**	2	7	9	20
**Site locations**	Northern NSW, Southern QLD		Northern NSW, southern and central QLD	National

*Multi-environment yield trial, **national variety trial.

### Crop Safety Evaluation

In 2020, dryland field trials to assess crop safety of D15PAHI002, D16PAHI001, and CBA2061 against cv. PBA HatTrick were conducted at two sites, Riverton and Turretfield, South Australia. At both sites, field trials were arranged in a RCBD with three blocks. Herbicide treatments were applied at the 5-node growth stage using a shrouded plot sprayer with flat fan nozzles at a 100 L spray volume ha^–1^ applied at 1 ms^–1^ and 220 kPa.

At Riverton, Trial 1 compared the response to application at the 5-node stage of 0, 0.5, 1, and 2 times the field use rate of 24.8 g ha^–1^ of imazamox + 11.3 g ha^–1^ of imazapyr recommended for imidazolinone-tolerant wheat (*Triticum aestivum* L.), faba bean (*Vicia faba* L.), and lentil. Trial 2 compared the response to simulated herbicide residue conditions by “incorporated by sowing” (IBS) treatments of 4.2 g a.i. ha^–1^ of metsulfuron methyl (Ally ^®^ 600 g kg^–1^ of metsulfuron-methyl FMC Australia) and 9 g a.i. ha^–1^ of chlorsulfuron (Glean ^®^). At Turretfield, the response of CBA2061 and cv. PBA HatTrick were compared at 0, 1, and 2 times the field use rate of 24.8 g ha^–1^ of imazamox + 11.3 g ha^–1^ of imazapyr applied at the 5-node growth stage.

Herbicide damage scores were based on degree of chlorosis and plant stunting per plot and were taken at maximum herbicide expression *viz*. 8 weeks after treatment for post-emergent herbicide application and 16 weeks after treatment for IBS herbicide application. Grain yield measurements were recorded at physiological maturity. Results were analyzed using linear mixed models with the ASReml package (Butler et al., 2018). Additional site-specific extraneous fixed and random terms were included as required, and residual errors for each site were modeled using spatial methods. Residual maximum likelihood (REML) methodology was used for variance parameter estimation.

### Factor Analytic Linear Mixed Model Statistical Approach for Multi-Environment Trial Evaluation

The approach of Smith et al. (2021a) was used to construct a MET dataset designed to maximize the amount of direct data on genotypes under consideration for selection in the current year (Cullis et al., 2020). Rather than generating separate designs for each trial, we implemented a new class of design, suited to Stage 1 (S1) trials. These designs are incomplete MET (IMET) designs. A comprehensive account of these methods is in preparation. Early versions of the ideas have been presented^3^. All designs were generated using the R package (R Core Team, 2020) OD (Butler and Cullis, 2018).

## Results

### Mutation and Selection of Herbicide-Tolerant Plant Material

Following the mutagenesis of cv. PBA HatTrick with EMS, field screening across a 20 ha site for tolerance to Group 2 herbicide imazapyr resulted in the identification and confirmation of 14 putative tolerant selections. In 2015, M_4_ seeds were harvested from 13 of the 14 selections. Progeny testing confirmed putative tolerance of one M_5_ mutant line, D15PAHI002, and this line was provided to NSW DPI in 2016 and entered the accelerated breeding timeline (Figure 1). At 21 DAT with imazapyr, the 12 other selections were found to be severely damaged and did not yield seed.

In 2016, a 1 ha field screen for tolerance to imazapyr and imazapic, a common herbicide mix used in Australia, resulted in five further putative Group 2-tolerant selections. Of the three selections that yielded M_3_ seeds and were progeny-tested, one M_4_ line, D16PAH001, progressed to further evaluation. The remaining selections were severely damaged and did not yield seed.

**FIGURE 2:**
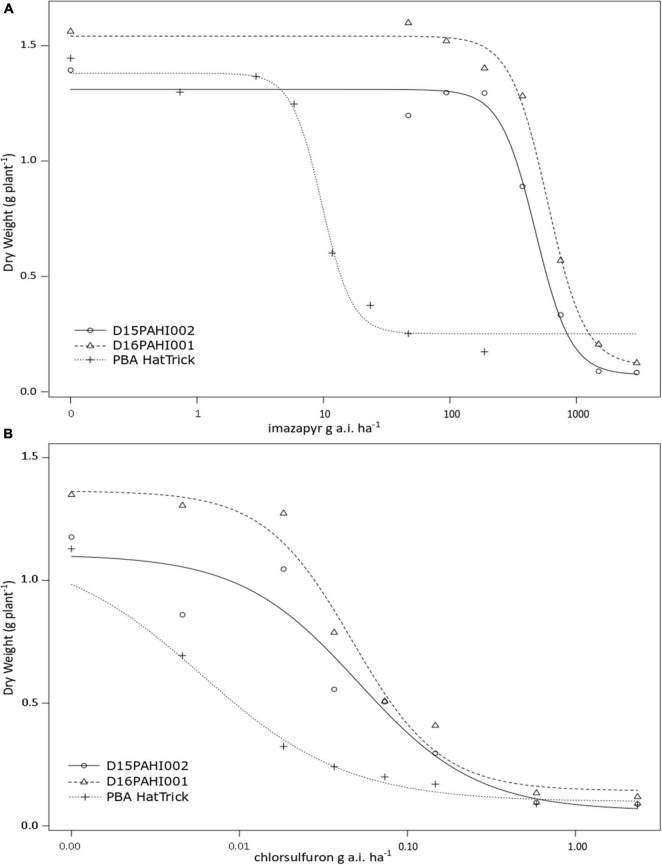
Plant dry weight response at 21 days after treatment to increasing rates of imazapyr **(A)** or chlorsulfuron **(B)** of two mutant lines D15PAHI002 and D16PAHI001 and reference check; the mutant’s background cv. PBA HatTrick.

In-pot controlled environment dose response trials demonstrated the mutant lines D15PAHI002 and D16PAHI001 and cultivar PBA HatTrick had typical herbicide response curves based on dry weight response to increasing rates of imazapyr and chlorsulfuron (Figures 2A,B). The mutant lines had a high level of tolerance to imazapyr. In response to imazapyr, the resistance factor (RF) for D16PAHI001 was significantly higher than for D15PAHI002 (Table 4). However, resistance of both was far higher, 51-fold and 39-fold, respectively, than cv. PBA HatTrick. Overall tolerance of both lines to chlorsulfuron was four to five times lower than tolerance to imazapyr (Table 4).

**TABLE 4:** Estimated parameters – including resistance factor (RF) and 50% growth reduction (GR_50_) values comparing the response of two mutant lines D15PAHI002 and D16PAHI001 to control cv. PBA HatTrick to the imidazolinone herbicide imazapyr or sulfonylurea herbicide chlorsulfuron.

Herbicide family	Active ingredient	Biotype	Upper limit	Slope	GR_50_	RF
Imidazolinone	Imazapyr	PBA HatTrick	1.83 (0.52) ^‡^	1.39 (0.07) ^‡^	11.38 (2.14) ^‡^	–
		D15PAHI002	3.11 (0.92) ^†^	1.31 (0.06) ^‡^	476.46 (59.43) ^‡^	41.89 (9.45) ^‡^
		D16PAHI001	2.95 (0.94) ^†^	1.54 (0.06) ^‡^	590.11 (64.29) ^‡^	51.88 (11.27) ^‡^
Sulfonylurea	Chlorsulfuron	PBA HatTrick	1.00 (0.44) ^†^	1.13 (0.10) ^‡^	0.01 (0.00) ^†^	–
		D15PAHI002	1.23 (0.60) ^†^	1.11 (0.09) ^‡^	0.05 (0.02) ^‡^	8.56 (4.44) *
		D16PAHI001	1.66 (0.50) ^‡^	1.36 (0.07) ^‡^	0.05 (0.01) ^‡^	7.8 (3.56) *

Data were analyzed in the DRC package in R Studio. SE for parameter estimates in parentheses, GR_50_ is the herbicide application rate required to reduce the response of plants to 50%, RF is the resistance factor (GR50 mutant/GR50 control cultivar). Level of significance: *P = 0.10, ^†^P = 0.05, ^‡^P = 0.01.

### Crop Safety Evaluation

**FIGURE 3:**
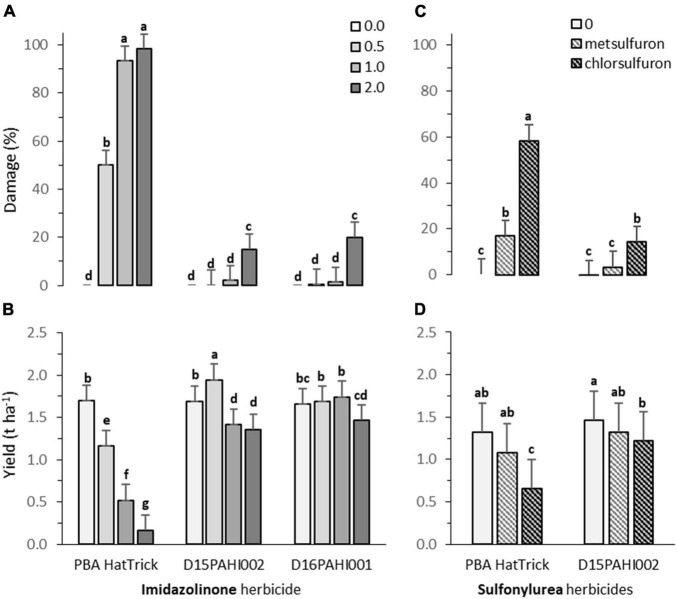
Plant damage (%) and yield (t ha^– 1^) for cultivar PBA HatTrick and derivative mutants DH15PAHI002 and D16PAHI001 selected for tolerance to Group 2 imidazole herbicide following **(A,B)** post-emergent (5-node stage) applications of herbicides, at 0, 0.5, 1.0, and 2.0 times the recommended field use rate of 24.8 g a.i. ha^– 1^ of imazamox + 11.3 g a.i. of imazapyr; and **(C,D)** for line DH15PAHI002 and PBA HatTrick, incorporated by sowing (IBS) applications of two Group 2 sulfonylurea herbicides at their recommended rate, 4.2 g a.i. ha^– 1^ of metsulfuron and 9.0 g a.i. ha^– 1^ of chlorsulfuron, in the 2020 trials at Riverton, South Australia. Plant damage was scored at 8 weeks after post-emergence spray treatment **(A)** or 16 weeks following herbicide incorporated by sowing (IBS) treatment **(C)**. In each graph, bars indicate means and whiskers indicate LSD (α = 0.05); different letters (a-g) represent significantly different means.

Lines D15PAHI002 and D16PAHI002 exhibited a high level of imidazolinone tolerance and no damage at the recommended field application rate prescribed for other AHAS inhibitor-tolerant crops (24.8 g of imazamox + 11.3 g of imazapyr ha^–1^, Figure 3A). Line D15PAHI002 had *c*. 20% yield reduction at this recommended rate and yield was not further reduced when the rate was doubled. Line D16PAHI001 had equivalent yields in the control treatment and at 2× the recommended rate, *c*. 11% less than for the 0.5× and 1× rates (Figure 3B). By comparison, cv. PBA HatTrick exhibited high levels of damage at both the 1× and 2× field rates with c. 70% yield reduction at the 1× rate and >90% yield reduction at the 2× rate (Figures 3A,B). In the current 2021 season, further crop safety field trials are underway at three locations to confirm this identified level of field HT has transferred to CBA2061.

Following sowing into simulated residues of metsulfuron herbicide, no significant plant damage was observed 16 weeks after sowing in line D15PAHI002 compared with 17% damage observed in PBA HatTrick (Figure 3C). For chlorsulfuron residues, moderate damage of *c*. 14% was observed in D15PAHI002 in contrast to *c*. 58% in PBA HatTrick (Figure 3C). The metsulfuron application rate of 4.2 g a.i. ha^−1^ was insufficient to cause yield reduction in either genotype under the seasonal and soil conditions for this site (Figure 3D). However, for chlorsulfuron residue treatment, yield was moderately reduced for D15PAHI002 (av. 1.5 to 1.2 t ha^−1^) and substantially reduced (av. 1.3 to 0.7 t ha^−1^) for PBA HatTrick (Figure 3D).

### Trait Introgression and Acceleration

Line D15PAHI002 was the first putative HT selection to be identified by SARDI and was crossed opportunistically by NSW DPI with cv. PBA Seamer in 2016 (Table 1). PBA Seamer was selected for its semi-erect plant type, wide adaptation, good yield stability, and moderate resistance to Ascochyta blight and Phytophthora root rot. Hybrid seeds from this cross and two others, D15PAHI002 × D1007 > 11F2TMWR2SS007 and D15PAHI002 × K15195 > F103, were grown in the glasshouse at NSW DPI and split into two sets.

Grown under aSSD conditions, the first set of F_2_ seeds (*n* = 186) flowered within 23–28 day of sowing and immature seeds were removed at 18 days after flowering. Taking T_*b*_ to be 0°C for chickpea (Lake et al., 2016), growing degree days from sowing to harvest ranged within 874 to 981 days. Following MAS, a total of 43 lines were recorded as homozygous for the HT trait and were resown to the following generation. The F_4_ seeds were left to fully mature on the plant and 7–24 seeds from each line were returned to NSW DPI on July 23, 2017 for further multiplication and evaluation. The total generation cycle time was *c*. 60 days for F_2_ and *c*. 72 days for F_3_ to allow for seed maturation on the mother plant.

The second set of F_2_ seeds (*n* = 790) was grown and evaluated for Group 2 HT in a temperature-controlled plastic house at NSW DPI. Within the perlite screen, herbicide-susceptible F_2_ individuals and checks displayed yellowing or wilting 48 h after herbicide submergence treatment. The surviving plants (*c*. 1–35% of the F_2_ seedlings from each population) were transplanted and grown to maturity. This alternative pathway took two and a half years longer than the aSSD MAS pathway, with field-based single plant selections at F_3_ and single row multiplication required prior to yield trial entry.

Full-length coding sequences and translated amino acid sequences were generated for the *CaAHAS1* gene length for the tolerant D15PAHI002 line (GenBank accession OK078878) and the PBA HatTrick cultivar (GenBank accession OK078877). The D15PAHI002 and D16PAHI001 single amino acid substitution from alanine to valine at position 205 (A_205_V) is identical to that described by Thompson and Tar’an (2014) (NCBI ref. seq. XM_004501646.3). KASP™ markers were developed for the SNP and used to genotype the aSSD and perlite-selected F_2_ seedling sets. Among the F_2_ HT plants selected at NSW DPI, 46% were homozygous for the *CaAHAS1-*tolerant allele, the rest were heterozygous. At UWA, following MAS, only homozygous *CaAHAS1*-tolerant plants were processed to F_4_ resulting in substantial cost and time saving.

### Herbicide Trait Confirmation

The line D15PAHI002 and the 14 aSSD-derived D15PAHI002 × PBA Seamer progeny lines including CBA2061, previously confirmed to be homozygous for the *CaAHAS1-*tolerant allele, were visually assessed in-crop and exhibited no sign of herbicide damage. The non-tolerant check cultivars Genesis090, PBA Magnus, PBA Seamer, and PBA Slasher showed symptoms of severe herbicide damage. The aSSD-derived lines were hand-harvested, threshed, and seed-utilized for further field evaluations.

### Field Evaluation of Advanced Breeding Lines

**FIGURE 4:**
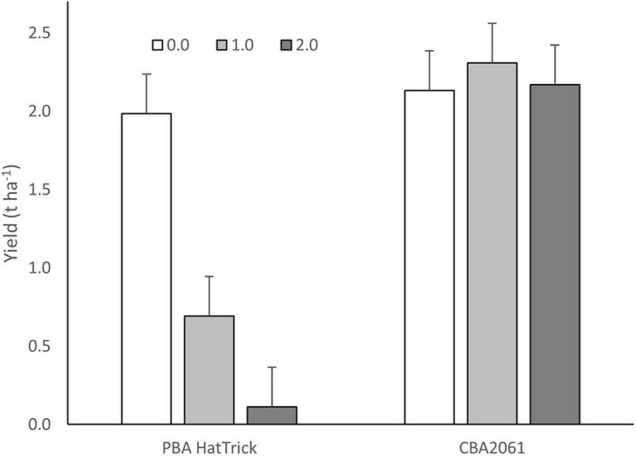
Yield response of CBA2061 compared to cv. PBA HatTrick following application of the recommended field use rate of 24.8 g a.i. ha^– 1^ of imazamox + 11.3 g a.i. ha^– 1^ of imazapyr and twice the recommended rate in the 2020 field trial at Turretfield, SA. Bars indicate means and whiskers indicate LSD (α = 0.05).

Following treatment with 49.5 g ha^–1^ of imazamox + 22.5 g ha^–1^ of imazapyr, twice the recommended field use rate, CBA2061 gave an equivalent yield to untreated PBA HatTrick (Figure 4). Plants began exhibiting herbicide effects 2 weeks after treatment with maximum herbicide expression at 6 weeks and plant recovery at 8 weeks. By harvest, CBA2061 had recovered from any herbicide effect and showed no yield reduction regardless of herbicide application rate. The yield of PBA HatTrick was reduced by 68% at the single rate herbicide application and almost to nil at the double rate.

Multi-environment trial evaluation of CBA2061 against five locally adapted cultivars demonstrated their agronomic characteristics were similar in the northern growing region (southern QLD and northern NSW). CBA2061 is an early flowering line with mid-season maturity suited to the winter farming system requirements of this growing region. The line has a semi-erect plant type with sufficient plant height and lodging tolerance to indicate good harvestability. Disease data showed a moderate and acceptable level of resistance to Ascochyta blight and Phytophthora root rot comparable to conventional cultivars PBA HatTrick and PBA Seamer. CBA2061 is an angular-shaped desi chickpea with medium sized Jimbour-type seed and good milling quality similar to PBA HatTrick and PBA Seamer.

### Field Evaluation of Yield Performance

The FALMM was fitted in a one-stage approach to permit model validation and examination in a statistically rigorous manner (as per Lee et al., 2006). FALMM was fitted to the desi north 2020 MET dataset along with an example of the application of the interaction class methodology for summarizing the results of the model fit (Smith et al., 2021b). There was a total of 55 environments and 99 trials in this MET dataset. Ancestral information was available on 5530 genotypes, with 4882 genotypes in the dataset. The mean inbreeding coefficient of the genotypes with data was 0.7621. A factor analytic (FA) model of order two and one was fitted to the additive GEI effects and the non-additive GEI effects, respectively. The percentage variance accounted for by the fit of the FA (2, 1) model was 77.9% with each of the three factors accounting for 35.7, 28.0, and 14.2%, respectively.

**FIGURE 5:**
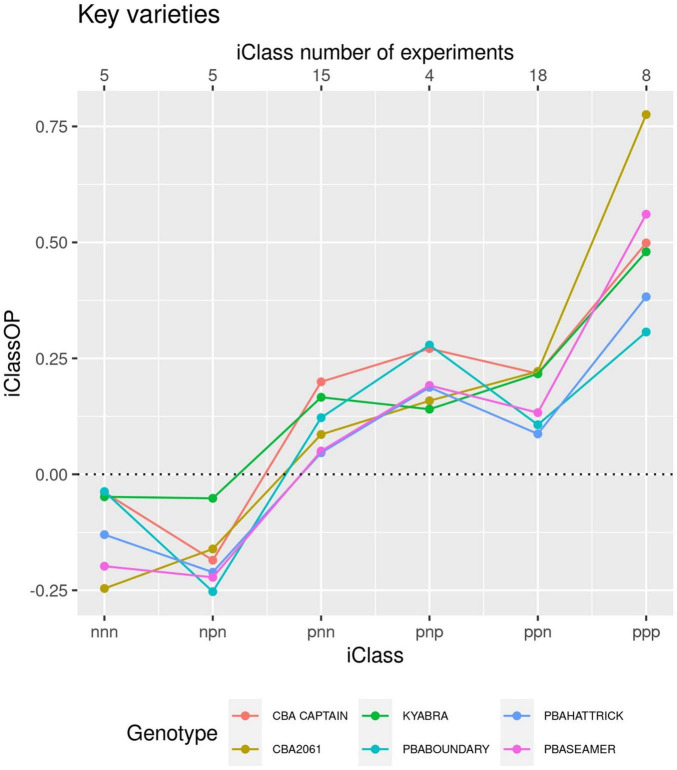
iClass interaction plot for the breeding line CBA2061 and comparison with five regionally adapted cultivars.

To obtain a graphical display of the crossover GEI in the MET dataset, a set of iClasses was created (as per Smith et al., 2021b) from the concatenated set of two-level factors, one for each factor and each with two levels [positive (p) and negative (n)], representing the sign of the loadings. For the desi dataset there were eight iClasses which formed the set of environments for which there was minimal crossover GEI between environments within the same iClass. The overall performance (OP) of each variety was the average of the E-BLUPs of the common variety effects from the latent FA regression model. The iClass Interaction Plot provides a metric for ordering the crossover GEI, as adjacent pairs of iClasses differ in the highest order factor so reflect the least amount of between iClass crossover GEI (Figure 5). Moving across iClasses from left to right in Figure 5 demonstrates the lack of GEI amongst the highly related (except for Kyabra) genotypes. All related genotypes had a very high OP with common parents and grandparents. Kyabra exhibited a slightly different GEI to the other genotypes, while the breeding line CBA2061 had almost an identical pattern of GEI to PBA Seamer but had a slight yield advantage in iClasses “ppn” and “ppp.”

## Discussion

A conventional, self-pollinated species breeding approach combined with aSSD, MAS, and sparse phenotyping platforms facilitated progression in 4 years (2016–2020) of the case study herbicide-tolerant chickpea genotype CBA2061 from cross to National Variety Trials (NVT). It is expected that cultivar release will occur 3.5 years earlier than through a conventional breeding pipeline (Figure 1). The accelerated approach facilitated rapid introgression of the mutation event Ala_205_Val into well-adapted cv. PBA Seamer and accelerated advancement of the resulting Group 2-tolerant genotype CBA2061. Subject to successful registration with chemical companies, CBA2061 will be an important rotation tool in the cropping system to control broadleaf weeds and enable the diversification of broadleaf herbicide groups across the Australian farming system. The Ala_205_Val mutation, as previously described in a Canadian chickpea genotype by Thompson and Tar’an (2014), confers high-level tolerance to the imidazolinone herbicides in-crop and moderate level tolerance to soil residues of the sulfonylurea herbicides. The mutant-derived HT advanced breeding line CBA2061 exhibits high levels of crop safety with no damage or yield reduction at 1× or 2× the recommended field use rate of the imidazolinone herbicides. As a result, the crop is expected to be extended into areas where difficult to control weeds cause issues and will diversify herbicide options and reduce the risk of both herbicide resistance in weeds and residual herbicide damage.

Mutating an elite, high-yielding background (cv. PBA HatTrick) was a key part of the breeding strategy to ensure any advantageous mutation would be within an appropriate and current adaptive background for the target production regions. The alternative HT introgression pathways of crossing locally adapted material with previously identified HT Canadian chickpea material (Thompson and Tar’an, 2014), or mutation of a local, but less adapted parental background, would have led to further crossing and evaluation stages and extended the breeding pipeline timeframe. Further, the strategy of crossing putative HT lines immediately after field selection while controlled environment and field HT confirmation experiments were still underway resulted in derivatives being available at the time of HT trait confirmation for D15PAHI002. Breeding resources were then preferentially allocated to ensure rapid progression of the D15PAHI002 × PBA Seamer progeny through the pipeline. This contrasts with the conventional approach of progeny screening, dose response experiments and field validation research undertaken separately for up to 2 years prior to providing germplasm and knowledge to breeders. The second mutation line identified in 2016, D16PAHI001, had the same mutation but gave higher yield in HT crop safety trials. This line has also been crossed with regionally adapted breeding material and its derivatives are currently under evaluation within the accelerated pipeline. The coordination between pre-breeding and breeding partners aimed at rapid introgression of this HT trait stands as an example of how publicly funded organizations can work together to improve the rate of genetic gain in a time efficient manner for a critical industry trait.

Among parameters in the breeder’s equation, cycle time is the easiest to understand, cheapest to manipulate, and the most powerful parameter for increasing genetic gain (Cobb et al., 2019). Cycle time, or generation interval, involves recycling breeding material back into the crossing block as quickly as a breeder can determine that a genotype is above average in breeding value for a desired quantitative trait. Rapid generation advancement, widely known as “speed breeding” (Watson et al., 2018), has been predominantly reported in Fabaceae species for recombinant inbred line production for molecular mapping and QTL discovery (Lulsdorf and Banniza, 2018; Uz Zaman et al., 2019; Atieno et al., 2021; Dadu et al., 2021; Taylor et al., 2021). Another practical use of accelerated life cycling is to achieve rapid out-of-season turnover of promising breeding germplasm. Cycling from F_2:4_ out of season gives breeders access to germplasm that can be screened in-season and in GEI trials for beneficial agronomic traits, saving a year in a conventional breeding pipeline. One constraint is the low seed number resulting from intensive growth under controlled conditions, designed to prioritize reproductive growth over vegetative biomass. Using a FALMM approach enabled field selections based on predicted values, and adapting the MET designs for Stage 1 trials allowed lines to progress through yield evaluation despite limited seed availability.

Combining established MAS with emerging aSSD techniques provided further time-saving in the breeding pipeline. Integrating platforms such as aSSD and MAS with conventional breeding programs is not straightforward and requires plant breeder trust in quality assurance practices of the organizations handling the germplasm on their behalf and capacity available within platforms at time points suited to the breeding program. MAS allows for rapid, accurate, and cost-effective screening of large numbers of plants in breeding programs minimizing the use of herbicide bioassays. Combining molecular markers with markers and bioassays for other traits like disease resistance simplifies and accelerates the stacking of multiple traits. In this case study of HT chickpea, the MAS technology reduced the time from cross to cross allowing for the continual introgression of desirable traits into elite lines for improved agronomic fit and yield potential for industry. Implementing the marker assays at the F_2_ generation reduced the total number of lines in aSSD by 75%, and MAS of perlite screen survivors eliminated heterozygous “escapes” ensuring only homozygous tolerant cross progeny were progressed and the efficient allocation of downstream breeding program resources toward priority germplasm.

Our findings confirm the practical feasibility of combining pre-breeding approaches into a pipeline to achieve rapid genetic improvement for a qualitative trait, herbicide tolerance. We note that a quantitatively inherited trait may require modification of this approach to account for the complexity of minor gene controls, e.g., genetic selection. A conventional breeding approach (Figure 1) would take 7 years to achieve a similar outcome for this qualitative trait introgression through a bulk pedigree method with single plant selection at F_3/4_. Pulses receive far less support for pre-breeding efforts when compared with cereal crops. Pulse Breeding Australia (PBA), commissioned from 2006 to 2019, was a publicly funded breeding initiative of The Grains Research and Development Corporation, state-based Agriculture Departments and Universities that delivered chickpea, pea (*Pisum sativum* L.), lentil, faba bean, and lupin (*Lupinus angustifolius* L.) varieties to Australian growers. PBA linked pulse breeding programs with a network of pre-breeding projects, enabling efficient adoption of emerging germplasm and pre-breeding tools within breeding programs in a co-ordinated manner. This joint research venture facilitated relationships among involved institutions to speed up trait delivery from pre-breeding to industry. The pipeline presented is an excellent applied example of (1) the benefit of investment by industry and government in novel pre-breeding tools and (2) demonstrable integration of such tools to accelerate genetic gain and delivery of improved varieties to producers. To achieve the output of a HT chickpea in NVT after 4 years has required collaborators to share a willingness to take risks, communicate well, and exchange breeding material and expertise in a manner which acknowledges the research and efforts of all participants. We expect this breeding approach can and will be used to accelerate genetic gain in any self-pollinated species with access to similar technologies.

## Data Availability Statement

The raw data supporting the conclusions of this article will be made available by the authors, without undue reservation.

## Author Contributions

TS, LM, CP, KH, BC, JC, WE, and FR were chief investigators on the projects contributing data to this manuscript. DM, LM, DB, FR, ND, and SM undertook HT, aSSD, MAS, and data analysis of HT experiments. BC conceived and undertook statistics related to breeding program MET and FALMM. FO coordinated the pre-breeding and breeding projects providing data to this manuscript. JC, ND, DM, SM, KH, and JL wrote the first draft of the manuscript. All authors contributed to the manuscript revision and editing and approved the submitted version of the manuscript.

## Conflict of Interest

The authors declare that the research was conducted in the absence of any commercial or financial relationships that could be construed as a potential conflict of interest.

## Publisher’s Note

All claims expressed in this article are solely those of the authors and do not necessarily represent those of their affiliated organizations, or those of the publisher, the editors and the reviewers. Any product that may be evaluated in this article, or claim that may be made by its manufacturer, is not guaranteed or endorsed by the publisher.

## Footnotes

1
https://www.ipcc.ch/ar6-syr/

2
https://www.geneious.com

3
https://documents.uow.edu.au/content/groups/public/@web/@inf/@math/documents/doc/uow269164.pdf
